# Sleep Duration, Sedentary Behaviors, and Physical Activity across Weight Status in Hispanic Toddlers’ Participants of the WIC Program

**DOI:** 10.21767/2572-5394.100017

**Published:** 2016-09-26

**Authors:** José Molina, Kiara Amaro, Cynthia M Pérez, Cristina Palacios

**Affiliations:** 1 Nutrition Program, School of Public Health, Medical Sciences Campus, University of Puerto Rico; 2 Department of Biostatistics and Epidemiology, School of Public Health, Medical Sciences Campus, University of Puerto Rico

**Keywords:** Obesity, Toddlers, Sleep duration, Sedentary behaviors, Physical activity, WIC Program

## Abstract

**Objective:**

To describe physical activity, sedentary behaviors and sleep duration in toddlers’ participants of the WIC program in Puerto Rico and assess its association with excessive weight.

**Methods:**

This was a cross-sectional analysis of data gathered in the follow-up visit (12 months later) of a longitudinal study among toddlers 12–36 months old participants of the WIC program. In this follow-up visit, a Sleep, Sedentary Behaviors and Physical Activity Questionnaire was included.

**Results:**

From the 213 eligible participants for the follow-up visit, 76 completed the follow-up visit. Most were girls (52.6%), with median age 21 months and most were categorized as healthy weight (76.3%). In general, toddlers spent a total median of 142 min/d in sedentary behaviors, 300 min/d in physical activities and 690 min/d sleeping. There was a higher duration of physical activities among overweight/obese compared to healthy weights (p<0.05) but similar duration of sedentary behaviors and sleep by weight status (p>0.05). There was a greater proportion of overweight/obese toddlers meeting the screen time recommendation (88.9%) compared to healthy weight toddlers (62.1%; p<0.05). Also, there was a significant positive age-adjusted correlation between time spent in unstructured physical activity (R=0.23, p<0.05) with weight-for-length z-score. Infant weight status was not significantly correlated to parent’s perception or knowledge of physical activity or sleep in toddlers (p>0.05).

**Conclusion:**

Most toddlers studied met the recommendations for duration of sleep, sedentary behaviors, and physical activity. Overweight/obese toddlers engage in more physical activities than those with a healthy weight status. These findings could be due to educational interventions by the WIC program to promote physical activities, as these toddlers are active WIC participants.

## Introduction

Childhood obesity is a growing problem in both developed and under-developed countries [1]. By 2013, this global issue was affecting over 42 million of children under the age of five, of which, 31 million were living in developing countries, as reported by the World Health Organization [1]. In the US, 8.4% of children between two and five years old are obese [2]. This is higher among Hispanics (16.7%) compared to non-Hispanic whites in this same age group (3.5%). In addition, Hispanics experience greater weight gain during the first years of life compared to non-Hispanic whites [3]. Furthermore, among young children participating in the Special Supplemental Nutrition Program for Women, Infants, and Children (WIC) program, the overall prevalence of obesity is even higher compared to the general US population (16.1%) [4]. This is a public health issue that must be addressed, as there is plenty of evidence showing a direct relation between infant obesity and higher risk of obesity and cardio-metabolic diseases later in life [5,6].

Some studies link this excess weight with inadequate breastfeeding practices, early introduction of complementary foods, low diet quality, lack of physical activity, sedentary lifestyles, inadequate sleep duration, among others factors in young children [7–9]. However, there are limited studies evaluating the association between physical activity, sedentary lifestyles and sleep among toddlers (12–36 months). This is a critical period in which toddlers are experimenting important dietary transitions [10] and other lifestyle transitions in the family environment. In addition, to our knowledge, there are no studies evaluating these behaviors with weight status in Hispanics, a group with large health disparities and high burden of obesity and other chronic diseases [11] and among participants of the WIC program. The WIC program is particularly fundamental in preventing obesity early in life, as it provides supplemental foods, health care referrals, and nutrition education for low-income pregnant, breastfeeding, and non-breastfeeding postpartum women, and to infants and children up to age five in the US and territories. Low income children are precisely the group with the greatest early weight gain risk. About 50% of small children in the US participate in the WIC program, with a total of 8.3 million US participants in 2012 [12]. This participation rate is even higher in Puerto Rico, with about 79% of children participating in WIC-Puerto Rico [13]. Therefore, studies aiming at understanding factors associated with weight gain among WIC participants may help in designing effective interventions to prevent obesity among the majority of children in low-income families in the US and in Puerto Rico.

The primary objective of the present study was to describe physical activity, sedentary behaviors, and sleep duration in a sample of Hispanics toddlers’ participants of the WIC Program in Puerto Rico and assess its association with excessive weight. A secondary objective was to assess the association between parental or caregiver’s parental perceptions or knowledge of infant’s activity with excessive weight.

## Materials and Methods

### Study design

Data for the present study were drawn from the follow-up visit of a longitudinal study being conducted among infants and toddler participants of the Women, Infants, and Children (WIC) clinic of Trujillo Alto in Puerto Rico. The first visit was conducted among 296 infants and toddlers aged 0–24 months in 2014–2015. The inclusion criteria were caregivers of singleton infants and toddlers, aged 21 years or older, and participants of WIC. The only exclusion criterion was if caregivers had infants and toddlers with major anomalies and disabilities that could impede regular feeding practices. For this follow-up assessment, we included all children previously studied that consented to participate in future studies (n=213). This follow-up visit was performed one year later when toddlers were 12–36 months old. For the present analysis, we used data collected from the socio-demographic characteristics Questionnaire and from the Sleep, Sedentary Behaviors, and Physical Activities Questionnaire, which was only collected in the follow-up visit. We also assessed the child’s weight and length/height. The study was approved by the Institutional Review Board of the University of Puerto Rico-Medical Sciences Campus. Caregivers of toddlers’ participants of the WIC program provided written informed consent.

### Socio-demographic characteristics

This instrument included age and sex of the child. It also included caregiver’s age, education, relation to child, number of children at home, perception of toddler’s weight status and self-reported weight and height.

### Anthropometric measurements

Weight was measured in duplicates in pounds using a manual calibrated scale for infants and toddlers (Detecto, MO) while wearing light clothes, a clean diaper, and no shoes. Measurements were averaged and converted into kg. Recumbent length (cm) was obtained using an infant and toddlers stadiometer (Perspective Enterprices, MI) for those 12–24 months and height was obtained using a wall stadiometer for those 24–36 months. Measurements were taken in duplicates and the averaged. In order to assess weight status, weight-for-length z scores were calculated using the age- and sex-specific growth charts of the World Health Organization (WHO) [14]. Healthy weight status was defined as a weight-for-length z score between −2 and 2 standard deviations and overweight or obese was defined as a weight-for-length z scores ≥2 standard deviations [14].

### Sleep, sedentary behaviors and physical activities questionnaire

This instrument included questions related to the time and frequency the toddler spends sleeping, on sedentary behaviors, and on physical activities. This questionnaire was adapted from the questionnaires used in the “Prevention of overweight in infancy study” and “Project VIVA” [15,16]. It also included questions on parental or caregiver’s parental perceptions and knowledge about the recommendations for sleep, sedentary behaviors, and physical activity in toddlers. It was administered using a face-to-face interview and took about 20 minutes to complete. We assessed content validity of this questionnaire by having experts (Pediatrician, Exercise physiologist) review the relevance of the items.

The frequency and duration the toddler slept daily in the last 7 days was assessed. We divided the daily 24-hour period in three categories: morning naps (from 7:00 am to 12:00 pm), afternoon naps (from 12:00 pm to 8:00 pm), and night sleep (8:00 pm to 7:00 am). We multiplied the duration by the frequency reported to obtain the total time for each category. Then results from these categories were added to obtain the total sleep time per week and finally it was divided by 7 to obtain results per day. This value was compared to the recommendation of 11–13 hours of sleep per day proposed by the American Academy of Pediatrics [17].

We also assessed the frequency and time the toddler spent on sedentary behaviors in different settings, categorized as “restricted” (car seat, stroller, bouncer), “semi-restricted” (jumper or walker), and “less restricted” (play pen or play yards) in the last 7 days. The frequency and time spent watching T.V. or tablet/phone in the last 7 days was also examined. A similar approach to calculating total daily sleep time was used to obtain total sedentary time per day. This value was compared to the recommendation of <2 hours of sedentary behaviors per day proposed by the American Academy of Pediatrics [18].

For physical activities, we evaluated the frequency and time spent on structured (e.g., baby gym, swimming, and karate classes) and unstructured (e.g., home activities and playing with animals, friends, or parents) activities. A similar approach to calculating total daily sleep time was used to obtain total physical activity per day. This value was compared to the recommendation of >2 hours of physical activity per day proposed by the American Academy of Pediatrics [19].

### Statistical methods

Normality of the sample in this study was assessed using the Shapiro-Wilk test statistic. Weight-for-length z scores were not normally distributed; therefore, non-parametric tests were used. We used median and percentiles (25^th^ and 75^th^) and percentages to describe the study group. Mann Whitney U-test was used to compare duration of sleep, sedentary behavior, and physical activity by weight status (healthy and overweight/obese). Spearman correlations (crude and age-adjusted) were used to determine the correlations between weight-for-length z scores and duration of sleep, sedentary behaviors, and physical activity. Chi-square tests were conducted to examine the association between weight status and parental perceptions or knowledge of toddler’s activity and the association between weight status and compliance with recommendations for sleep, sedentary behaviors, and physical activity. Statistical analyses were performed using the SPSS program (version 17).

## Results

From the 213 eligible participants for the follow-up visit, 78 participants could not be reached, 13 refused to participate, 45 did not show up to the appointment and one had incomplete data. The final sample comprised 76 participants. As described in (Table 1), most caregivers were mothers, had a median age of 29 years, most completed any postsecondary education (67.1%) and had a median BMI of 26.8 kg/m2. There were no differences in these variables between toddlers classified as healthy weight or overweight/obese. However, there was a greater percentage of caregivers with overweight/obese toddlers that perceived their child as overweight (p<0.05). A total of 52.6% of the toddlers were girls, median age was 21 months, median weight-for-length z-score was 0.44, and most children were categorized as having a healthy weight (76.3%).

Results for time spent on sedentary behaviors, physical activities, and sleep in toddlers by weight status are shown in (Table 2). In general, toddlers spent a median of 60 minutes per day sitting in a restricted setting and about a median of 48 minutes per day watching screens (combined use of TV, tablets and phones) with a total median of 142 minutes per day in any sedentary behaviors. No significant differences were observed between healthy weight and overweight/obese toddlers for sedentary behaviors, although a trend for a lower duration of time spent using the tablet/phone in the overweight/obese group compared to the healthy group was observed (p=0.06). With respect to physical activities, toddlers spent a median of 300 minutes per day in unstructured activities. Time spent in physical activities was higher among overweight/obese compared to healthy weights (p<0.05). Most sleep time was during the night compared to during the morning or afternoon, with a total median sleep time of 690 minutes per day, with no differences by weight status (p>0.05). Compliance with the screen time recommendation was greater in overweight/obese toddlers (88.9%) compared to healthy weight toddlers (62.1%; p<0.05). However, compliance with the physical activity or sleep recommendation was similar between groups (p>0.05).

The correlations between time spent on sedentary behaviors, physical activity, and sleep are shown in (Table 3). Time spent in unstructured physical activity was significantly and positively correlated with weight-for-length z-score (unadjusted r=0.31, p<0.05; age-adjusted r=0.23, p<0.05). No significant correlation was observed between time sedentary time and time spent sleeping with weight-for-length z-score.

Parental or caregiver’s perception and knowledge about physical activity and sleep duration in toddlers is shown in Table 4. No significant associations were observed between any of these variables.

## Discussion

In this study we found that Hispanic toddlers’ participants of the WIC program spent a total median of 142 minutes in any sedentary behaviors, 300 minutes in unstructured physical activities and 690 minutes of sleep per day. Healthy weight and overweight/obese toddlers spent similar times engaged in sedentary behaviors and sleep but overweight/obese toddlers spent more time engaged in physical activities (p<0.05). Time spent in semi-restricted settings and in unstructured physical activities was significantly and positively correlated with weight-for-length z-score. Toddler weight status was not significantly correlated to parental perception or knowledge of physical activity or sleep in toddlers, but there was a greater proportion of overweight/obese toddlers meeting the screen time recommendation than healthy weight toddlers. Overall, most of the toddlers studied met the recommendations for duration of sleep, sedentary behaviors, and physical activity.

As mentioned above, most toddlers included in this study met the recommendation for sedentary time according to the American Academy of Pediatrics [20]. However, inconsistent with other studies [9,18], most of the overweight/obese toddlers did not exceed 2 hours of T.V. per day. In addition to T.V., overweight/obese toddlers did not engage more in other sedentary behaviors compared to healthy weight toddlers and overall, they did not have a sedentary lifestyle. As shown in other studies, educational interventions in young children, such as the intervention promoted by the WIC program to decrease sedentary behaviors and promote physical activities in young children are effective. For example, a parent focused intervention study showed how orientation sessions particularly focused on building knowledge and skills regarding physical activity and sedentary behaviors delivered by dietitians to parents can have a decrease in the time the toddlers spent watching T.V. [17]. Similarly, a WIC-based intervention study in California demonstrated that interventions in WIC have a positive impact in the health behaviors of toddlers aged 1 to 5 years old and suggests that results may extend to Latino populations [21]. Also, interventions including motivational Interviewing counseling strategies in WIC may help prevent childhood obesity [22]. These results suggest that the overweight/obese toddlers in this study were probably receiving active interventions by their WIC nutritionists to improve sedentary behaviors, as overweight/obese toddlers are considered at risk and are followed more frequently with more time spent on educational and intervention sessions with nutritionists at the clinics.

In the present study, most toddlers also met the physical activity recommendations according to the American Academy of Pediatrics [20]. Overweight and obese toddlers were more physically active per day than those with healthy weight, particularly in unstructured physical activities. This is contrary to other reports [9,23], in which most studies show a significant association between inactivity and childhood obesity. However, most studies have been conducted in children older than 3 years; little is known among younger children. The sample studied in the present study were active WIC participants; therefore, as mentioned earlier, our results could be explained by recent changes done at home by the mothers of overweight/obese toddlers to increase physical activity in response to recommendations from the WIC staff. Also, the association between physical activity and weight gain may not be clear during these years, as toddlers are experiencing rapid changes in their development and in their nutritional requirements [6]. Furthermore, these results could also be explained by an intermediate third variable, such as energy intake or diet quality. This should be furthered studied.

Decrease in sleep is becoming more common in children [24]. This was evidenced in the present study whereas approximately half the healthy weight and about 60% of overweight/obese toddlers did not meet the sleep recommendation according to the American Academy of Pediatrics [20]. However, there was no significant association between sleep time and weight status. This is contrary to results from a meta-analysis including 11 studies in children 0 to 19 years old, in which the shortest sleep duration was associated with higher risk of overweight/obesity (OR=1.92; 95% CI) [24]. This meta-analysis suggested that sleep deprivation (not having enough sleep time) affects weight among young children aged <10 years (trend test: P=0.094) and may be one of the main risk factors of overweight and obesity in children. However, the effects of poor sleep may not necessarily be manifesting yet in this early age group. Longer follow-ups may be needed to detect these effects.

The present study is one of very few that has taken into consideration the duration of sleep, sedentary behaviors, and physical activities and their associations with weight status in toddlers 12–36 months. However, some limitations should be addressed. Sedentarism, physical activity, and sleep time were assessed using a self-reported questionnaire, which is subject to error and bias. There are other factors associated with weight status, not only in toddlers but in the population in general that were not taken into account in the present study, such as dietary patterns, including breastfeeding, total energy intake, and other environmental and genetics factors. The sample size was small because only a third of the sample participated in the follow-up visit in which these measures were assessed; this could result in selection bias. The cross-sectional design of the study and the limited simple size does not allow us to infer causality. Longitudinal studies are needed with this population to determine the long-term associations between sleep, sedentary behaviors, and physical activities. In addition, the data was self-reported by the participants; therefore, there could be bias in the information collected. On the other hand, the strengths of this study include integration of factors related to sleep, sedentary behaviors, and physical activities for a more comprehensive analysis and can serve as an example to explore more deeply these factors specifically in this population.

In conclusion, most toddlers studied met the recommendations for duration of sleep, sedentary behaviors, and physical activity. A positive significant association was observed between unstructured physical activity and weight status, whereas those overweight and obese toddlers engage in more physical activities than those with a healthy weight status. These findings could be due to educational interventions by the WIC program to promote physical activities, as these toddlers are active WIC participants. Special emphasis should be given in sleep time, because half the sample did not meet the recommendations. Longitudinal studies are needed to understand how these factors are associated with weight change over time in this group of young children.

## Figures and Tables

**Table 1:** Socio-demographic characteristics of caregivers and their toddlers and weight status of toddlers (n=76).

Characteristics	Total sample (n=76)	Healthy weight (n=58)	Overweight/obese (n=18)	P value^1^
	Median (25^th^, 75^th^ percentiles) or Frequency % (N)			
**Caregivers**				
Age (y)	29.0 (24.2, 35.0)	29.0 (20.0, 72.0)	27.5 (23.0, 40.0)	0.89
**Sex**				
Female	98.7% (75)	98.3% (57)	100% (18)	0.68
Male	1.3% (1)	1.7% (1)	0	
**Relation to the baby**				
Mother	94.7% (72)	93.1% (54)	100% (18)	-
Father	1.3% (1)	1.7% (1)	0	0.69
Grandparent	4.0 (3)	5.2% (3)	0	-
**Education**				
≤ High school	32.9% (25)	31.1% (18)	38.9% (7)	-
>High school	67.1% (51)	68.9% (40)	61.1% (11)	-
Number of children at home	2.0 (1.0, 2.8)	2.0 (1.0, 5.0)	2.0 (1.0, 7.0)	0.55
**Caregiver perception of the toddler’s weight status**				
Underweight	3.97% (3)	5.2% (3)	0	-
Normal weight	86.8% (66)	91.4% (53)	72.2% (13)	0.04
Overweight	9.2% (7)	3.4% (2)	27.8% (5)	-
BMI (kg/m^2^)	26.8 (22.4, 29.7)	25.8 (16.5, 49.2)	27.4 (18.3, 38.3)	0.12
**Infants**				
**Sex**				
Girl	52.6% (40)	50.0% (29)	38.9% (7)	0.41
Boy	47.4% (36)	50.0% (29)	61.1% (11)	
Age (months)	21.0 (16.0, 28.0)	21.0 (11.0, 36.0)	21.0 (12.0, 35.0)	0.61
Weight/length percentile	67.0 (38.5, 92.0)	55.5 (7.0, 93.0)	97.0 (86.0, 100)	<0.01
Weight/length z score	0.44 (−0.29, 1.39)	0.32 (−1.51, 1.50)	2.29 (1.56, 5.26)	<0.01
**Weight status**				
Healthy weight	76.3% (58)	-	-	-
Overweight/obese	23.7% (18)	-	-	-

**Table 2:** Sedentary, physical activity, and sleep duration and compliance with their recommendations by weight status of toddlers.

Variable	Total sample (n=76)	Healthy weight (n=58)	Overweight/obese (n=18)	P value^1^
Duration	Median (25^th^, 75^th^ percentiles)			
**Sedentary activities (min/d)**				
Restricted settings^2^	60.0 (30.0, 90.0)	52.5 (30.0, 90.0)	60.0 (38.5, 120)	0.41
Semi-restricted settings^3^	0	0	0	-
Less restricted settings^4^	0	0	0	-
Watching T.V.	32.5 (20.0, 90.0)	30.0 (20.0, 94.5)	47.5 (20.0, 63.7)	0.79
Using tablet/phone	15.0 (0.00, 45.0)	17.5 (1.50, 60.0)	9.50 (0.0, 30.0)	0.06
Total	142 (90.0, 241)	142 (97.5, 270)	140 (78.0, 196)	0.42
**Physical activity (min/d)**				
Structured	0	0	0	-
Unstructured	300 (180, 360)	300 (180, 315)	330 (300, 540)	<0.05
Total	300 (180, 360)	300 (180, 327)	334 (300, 540)	<0.05
**Sleep (min/d)**				
Morning nap	17.0 (0.00, 60.0)	28.0 (0.0, 60.0)	0.0 (0.0, 42.0)	0.26
Afternoon nap	36.5 (9.00, 90.0)	52.5 (9.00, 90.0)	30.0 (8.25, 47.2)	0.1
Night time sleep	600 (540, 660)	600 (540, 660)	540 (525, 660)	0.34
Total	690 (591, 749)	690 (622, 753)	624 (539, 747)	0.16
**Compliance with recommendations % (N)**				
**Screen time**				
<2 hours^5^	68.4 (52)	62.1 (36)	88.9 (16)	0.03*
≥2 hours	31.6 (24)	37.9 (22)	11.1 (2)	
**Sleep time**				
11–13 hours^5^	48.7 (37)	55.2 (26)	38.9 (11)	0.22
<11 hours	51.3 (39)	44.8 (32)	61.1 (7)	
**Physical activity time**				
>2 hours^5^	92.1 (70)	94.8 (55)	83.3 (15)	0.11
≤2 hours	7.9 (6)	5.2 (3)	16.7 (3)	

1 P-value obtained using Mann Whitney U test or Chi square/Fisher exact Chi-square, as appropriate;

2 Restricted settings: sitting in a car set, stroller, bouncer chair, etc.;

3 Semi-restricted settings: jumper, walker, etc.;

4 Less restricted settings: play pen or play yard;

5 Recommendation from the American Academy of Pediatrics;

* p<0.05.

**Table 3:** Spearman correlations between sedentary time, physical activity, and sleep duration with weight-for-length z score of toddlers.

Variable	Correlation with weight/length z score	Age-adjusted correlation with weight/length z score
**Sedentary time (min/d)**		
Restricted settings^1^	0.14 (0.24)	0.03 (0.74)
Semi-restricted settings^2^	-	-
Less restricted settings^3^	-	-
Watching T.V.	0.01 (0.99)	−0.03 (0.75)
Watching others screens (tablet/phone)	0.02 (0.99)	−0.12 (0.30)
Total	−0.02 (0.88)	−0.04 (0.74)
**Physical activity (min/d)**		
Structured	-	-
Unstructured	0.31 (<0.05)*	0.23 (<0.05)*
Total	0.32 (<0.05)*	0.23 (<0.05)*
**Sleep time (min/d)**		
Morning nap	−0.02 (0.88)	0.01 (0.91)
Afternoon nap	−0.19 (0.10)	−0.19 (0.09)
Night time sleep	−0.07 (0.54)	−0.05 (0.65)
Total	−0.09 (0.43)	−0.10 (0.39)

1 Restricted settings: sitting in a car set, stroller, bouncer chair, etc.;

2 Semi-restricted settings: jumper, walker, etc.;

3 Less restricted settings: play pen or play yard;

* p<0.05.

**Table 4:** Association between parental perceptions and knowledge of physical activity and sleep duration in infants and toddlers according to weight status (% (N)).

Variables		Healthy weight N=58	Overweight/Obese N=18	P value
**Parent’s perceptions**				
Passive play is physical activity	Yes	69.0 (40)	66.7 (6)	0.85
	No	31.0 (18)	33.3 (12)	
Importance of physical activity in infants	Yes	100 (58)	100 (18)	-
	No	0	0	
Importance of being an active parent	Yes	100 (58)	100 (18)	-
	No	0	0	
**Parent’s knowledge**				
Physical activity recommendation for infants	Yes	10.3 (6)	0.00 (0)	0.15
	No	89.7 (52)	100 (18)	
Total sleep recommendation for infants	Yes	39.7 (23)	27.8 (5)	0.36
	No	60.3 (35)	72.2 (13)	

* p<0.05
